# Autonomous artificial intelligence for diabetic eye disease increases access and health equity in underserved populations

**DOI:** 10.1038/s41746-024-01197-3

**Published:** 2024-07-22

**Authors:** Jane J. Huang, Roomasa Channa, Risa M. Wolf, Yiwen Dong, Mavis Liang, Jiangxia Wang, Michael D. Abramoff, T. Y. Alvin Liu

**Affiliations:** 1 Wilmer Eye Institute, Johns Hopkins University School of Medicine, Baltimore, MD USA; 2 University of Wisconsin-Madison School of Medicine and Public Health, Madison, WI USA; 3 Johns Hopkins Pediatric Diabetes Center, Johns Hopkins University School of Medicine, Baltimore, MD USA; 4 Department of Biostatistics, Johns Hopkins University, Baltimore, MD USA; 5 Department of Ophthalmology and Visual Sciences, University of Iowa, Iowa City, IA USA; 6 Department of Electrical and Computer Engineering, University of Iowa, Iowa City, IA USA

**Keywords:** Retinal diseases, Disease prevention, Outcomes research, Diabetes complications, Machine learning

## Abstract

Diabetic eye disease (DED) is a leading cause of blindness in the world. Annual DED testing is recommended for adults with diabetes, but adherence to this guideline has historically been low. In 2020, Johns Hopkins Medicine (JHM) began deploying autonomous AI for DED testing. In this study, we aimed to determine whether autonomous AI implementation was associated with increased adherence to annual DED testing, and how this differed across patient populations. JHM primary care sites were categorized as “non-AI” (no autonomous AI deployment) or “AI-switched” (autonomous AI deployment by 2021). We conducted a propensity score weighting analysis to compare change in adherence rates from 2019 to 2021 between non-AI and AI-switched sites. Our study included all adult patients with diabetes (>17,000) managed within JHM and has three major findings. First, AI-switched sites experienced a 7.6 percentage point greater increase in DED testing than non-AI sites from 2019 to 2021 (*p* < 0.001). Second, the adherence rate for Black/African Americans increased by 12.2 percentage points within AI-switched sites but decreased by 0.6% points within non-AI sites (*p* < 0.001), suggesting that autonomous AI deployment improved access to retinal evaluation for historically disadvantaged populations. Third, autonomous AI is associated with improved health equity, e.g. the adherence rate gap between Asian Americans and Black/African Americans shrank from 15.6% in 2019 to 3.5% in 2021. In summary, our results from real-world deployment in a large integrated healthcare system suggest that autonomous AI is associated with improvement in overall DED testing adherence, patient access, and health equity.

## Introduction

Diabetic eye disease (DED) affects a third of people with diabetes mellitus (DM) and is a leading cause of blindness and visual impairment in working-aged adults in the developed world^1^. Since patients with DED often have no symptoms in the early stages of disease, current guidelines from the American Academy of Ophthalmology and American Diabetes Association recommend that patients with diabetes receive an annual eye examination^2,3^. These annual screenings allow for early diagnosis and treatment that can help prevent severe vision loss^4^. As such, annual DED testing is included as a Healthcare Effectiveness Data and Information Set (HEDIS) measure.

Unfortunately, adherence to these annual DED testing guidelines has historically been low. A previous study in the United States found that only 50–60% of Medicare beneficiaries with DM received annual eye exams^5^. This adherence rate is even lower (30–40%) in smaller health systems and low-income metropolitan patient populations^3,6^. Previous qualitative investigations have identified misinformation about the importance of regular testing, logistic challenges with scheduling appointments, and anxiety as key barriers to annual eye exams^7^. In 2018, the FDA De Novo authorized the use of an autonomous artificial intelligence (AI) system (LumineticsCore® formerly known as IDx-DR, Digital Diagnostics, Coralville, IA) to diagnose DED. This system can autonomously analyze images of the retina at the point of care, such as at primary care clinics, with a sensitivity of 87.2% and specificity of 90.7% in a pivotal clinical trial against a prognostic standard, i.e. patient outcome^8^. Subsequent studies have further validated the accuracy, sensitivity, and specificity of this autonomous AI technology, using ophthalmologists’ reads as the reference standard^9–11^.

In 2020, Johns Hopkins Medicine, an integrated healthcare system with over 30 primary care sites, began deploying autonomous AI for DED testing in some of its primary care clinics. This study aimed to determine whether implementation of this technology increased the rate of care gap closure in different patient populations and whether its implementation was associated with higher overall adherence rates.

## Results

### Patient demographics

A total of 17,674 patients with diabetes were managed at Johns Hopkins Medicine in 2019. Most patients were female (53.0%) and under 65 years old (69.0%). The two most highly represented racial groups were White (45.2%) and Black or African American (40.6%), and the two most common insurance coverages were commercial/other (48.7%) and Medicare (30.0%). Nearly all patients resided in an urban setting.

A total of 17,590 patients with diabetes were managed at Johns Hopkins Medicine in 2021. Again, most patients were female (51.1%) and under 65 years old (71.1%). Most patients were White (47.9%) and Black or African American (37.1%), and most patients had either commercial/other insurance (53.1%) or Medicare (28.1%). A detailed breakdown of the population demographics is available in Table 1. Overall, the patient demographics between AI-switched sites and non-AI sites were similar. Patients at non-AI sites had a higher inflation-adjusted mean income ($90,200 in 2019 and $98,000 in 2021) than patients at AI-switched sites ($63,400 in 2019 and $63,400 in 2021). Additionally, more patients at non-AI sites were covered under military insurance compared to AI sites (13.0% vs. 6.2%).

**Table 1:** Baseline patient demographics of AI-switched sites and non-AI sites in 2019 and 2021

	2019			2021		
*N* (%)	AI-switched sites	Non-AI sites	*P*-value	AI-switched sites	Non-AI sites	*P*-value
*Total patients*	5505	12169		5580	12010	
*Gender*			<0.001			<0.001
Female	3037 (55.2)	6334 (52.1)		3040 (54.5)	5989 (49.9)	
Male	2468 (44.8)	5835 (47.9)		2540 (45.5)	6021 (50.1)	
*Age*			0.015			0.929
Under 65 years	3730 (67.8)	8470 (69.6)		3982 (71.4)	8580 (71.4)	
65 years and over	1775 (32.2)	3699 (30.4)		1598 (28.6)	3430 (28.6)	
*Race*			<0.001			<0.001
Black or African American	2606 (47.3)	4563 (37.5)		2648 (47.5)	3907 (32.5)	
White	2411 (43.8)	5581 (45.9)		2402 (43.0)	5964 (49.7)	
Other	258 (4.7)	1150 (9.5)		288 (5.2)	1121 (9.3)	
Asian	190 (3.5)	783 (6.4)		202 (3.6)	922 (7.7)	
American Indian or Alaska Native	25 (0.4)	64 (0.5)		27 (0.5)	67 (0.6)	
Native Hawaiian or Other Pacific Islander	15 (0.3)	28 (0.2)		13 (0.2)	29 (0.2)	
*Ethnicity*			<0.001			<0.001
Not Hispanic or Latino	5253 (95.4)	11108 (91.3)		5297 (94.9)	11024 (91.8)	
Hispanic or Latino	139 (2.5)	672 (5.5)		159 (2.9)	594 (5.0)	
Unknown	113 (2.1)	389 (3.2)		124 (2.2)	392 (3.2)	
*Language Preference*			<0.001			0.177
English	5393 (98.0)	11679 (96.0)		5450 (97.7)	11687 (97.3)	
Non-English	112 (2.0)	490 (4.0)		130 (2.3)	323 (2.7)	
*Insurance Coverage*			<0.001			<0.001
Commercial and Other	2823 (51.3)	5780 (47.5)		2977 (53.4)	6408 (53.4)	
Pure Medicare	1738 (31.6)	3554 (29.2)		1630 (29.2)	3336 (27.8)	
Military	339 (6.1)	1584 (13.0)		325 (5.8)	1585 (13.2)	
Medicaid	295 (5.4)	646 (5.3)		329 (5.9)	332 (2.8)	
Medicare Advantage	246 (4.5)	285 (2.4)		243 (4.3)	244 (2.0)	
Self-Pay	64 (1.1)	320 (2.6)		76 (1.4)	105 (0.8)	
*Geography*			0.130			0.405
Metropolitan	5494 (99.8)	12147 (99.8)		5569 (99.8)	11990 (99.8)	
Micropolitan	10 (0.2)	11 (0.1)		10 (0.2)	13 (0.1)	
*National ADI*			<0.001			<0.001
1st Quartile (1–25)	417 (7.6)	3767 (31.0)		430 (7.7)	4298 (35.8)	
2nd Quartile (26–50)	943 (17.1)	3453 (28.3)		958 (17.2)	3812 (31.7)	
3rd Quartile (51–75)	1973 (35.8)	2553 (21.0)		2015 (36.1)	2524 (21.0)	
4th Quartile (76–100)	2172 (39.5)	2396 (19.7)		2177 (39.0)	1376 (11.5)	

*P*-values were generated from intra-year comparisons between AI-switched and non-AI sites.

### Propensity score weighting analysis

In our propensity score weighting analysis, we used inverse probability to assign weights to patients based on social determinants of health to ensure comparability across the different types of sites and years. The analysis showed that DED testing adherence among AI-switched sites increased by 7.4 percentage points (95% CI: [5.2, 9.5], *p* < 0.001) from 2019 to 2021. On the other hand, the adherence rate among non-AI sites decreased by 0.3 percentage points (95% CI: [−1.5, 0.9], *p* < 0.653). The change in adherence rate at AI-switched sites was 7.6 percentage points (95% CI [5.2, 10.1], *p* < 0.001) higher than that at non-AI sites, suggesting that there was a significantly higher increase in DED testing between 2019 and 2021 at AI-switched sites than at non-AI sites.

### Subgroup changes in DED adherence rate from 2019 to 2021

In 2019, the overall adherence rate across all sites was 42.2%. The baseline adherence rate was 46.1% at AI-switched sites and 40.4% at non-AI sites. In 2021, the overall adherence rate across all sites increased to 44.8%. Of note, the adherence rate increased to 54.5% at AI-switched sites and remained stable at 40.3% at non-AI sites. A detailed breakdown in adherence rate for each patient population is shown in Table 2.

**Table 2:** Change in patient adherence rate from 2019 to 2021 by demographic subgroups

	2019		2021		Change from 2019 to 2021		
	AI-switched sites	Non-AI sites	AI-switched sites	Non-AI sites	AI-switched sites [95% CI]	Non-AI sites [95% CI]	*P*-value
*Overall Adherence Rate*	46.1%	40.4%	54.5%	40.3%	+8.8% [+7.1%, +10.5%]	+0.3% [−0.8%, +1.4%]	**<0.001**
*Gender*							
Male	44.6%	39.9%	54.8%	39.6%	+10.5% [+7.9%, +13.1%]	+0.3% [−1.3%, +1.8%]	**<0.001**
Female	47.3%	41.0%	54.3%	40.9%	+7.4% [+5.1%, +9.7%]	+0.5% [−1.1%, +2.0%]	**<0.001**
*Age*							
65 years and over	57.1%	52.6%	64.6%	49.9%	+7.8% [+4.7%, +10.8%]	−2.1% [−4.2%, +0.0%]	**<0.001**
Under 65 years	40.9%	35.1%	50.5%	36.4%	+9.8% [+7.8%, +11.9%]	+1.6% [+0.3%, +2.9%]	**<0.001**
*Race*							
American Indian or Alaska Native	64.0%	39.1%	66.7%	43.3%	+2.3% [−20.7%, +25.2%]	+4.2% [−11.1%, +19.6%]	0.888
Asian	61.1%	43.4%	60.9%	41.3%	+0.4% [−8.7%, +9.5%]	-1.1% [−5.3%, +3.2%]	0.772
Black or African American	45.5%	40.9%	57.4%	39.7%	+12.2% [+9.7%, +14.7%]	−0.6% [−2.5%, +1.3%]	**<0.001**
Native Hawaiian or Other Pacific Islander	26.7%	50.0%	46.2%	48.3%	+19.0% [−17.9%, +55.9%]	−1.0% [-16.0%, +13.9%]	0.324
White	45.7%	40.5%	51.0%	40.4%	+5.8% [+3.2%, +8.3%]	+0.3% [−1.3%, +1.8%]	**<0.001**
Other	45.0%	36.3%	52.4%	40.5%	+8.1% [+0.1%, +16.0%]	+4.6% [+0.9%, +8.3%]	0.441
*Ethnicity*							
Hispanic or Latino	46.8%	35.7%	49.1%	38.6%	+2.7% [−7.2%, +12.7%]	+3.8% [−1.2%, +8.7%]	0.858
Not Hispanic or Latino	46.3%	40.7%	54.8%	40.6%	+8.9% [+7.1%, +10.6%]	+0.4% [−0.7%, +1.6%]	**<0.001**
Unknown	38.1%	42.4%	51.6%	34.2%	+14.5% [+3.0%, +25.9%]	−7.8% [−14.1%, -1.4%]	**<0.001**
*Language Preference*							
English	46.1%	40.6%	54.7%	40.3%	+8.9% [+7.2%, +10.7%]	+0.3% [-0.8%, +1.4%]	**<0.001**
Non-English	45.5%	37.1%	47.7%	37.5%	+2.8% [−9.1%, +14.7%]	+0.7% [−5.8%, +7.1%]	0.752
*Insurance Coverage*							
Pure Medicare	49.9%	43.9%	58.0%	42.3%	+8.5% [+5.5%, +11.6%]	−0.8% [-2.9%, +1.3%]	**<0.001**
Medicare Advantage	50.4%	53.7%	60.5%	46.3%	+9.8% [+1.7%, +17.9%]	−7.2% [−15.4%, +0.9%]	**0.004**
Medicaid	30.2%	36.2%	43.8%	31.6%	+13.7% [+6.7%, +20.7%]	−4.9% [−10.7%, +1.0%]	**<0.001**
Military	63.1%	50.8%	64.9%	50.0%	+2.8% [−4.2%, +9.8%]	−0.1% [-3.2%, +3.0%]	0.455
Self-Pay	42.2%	37.5%	44.7%	37.1%	+4.3% [−10.6%, +19.1%]	−0.8% [−10.5%, +9.0%]	0.580
Commercial and Other	43.1%	35.5%	52.5%	37.0%	+9.6% [+7.2%, +11.9%]	+1.9% [+0.4%, +3.5%]	**<0.001**
*Geography*							
Metropolitan	46.1%	40.5%	54.5%	40.3%	+8.7% [+7.0%, +10.4%]	+0.3% [-0.8%, +1.4%]	**<0.001**
Micropolitan	30.0%	27.3%	91.0%	23.1%	+59.8% [+27.1%, +92.4%]	−1.7% [-29.0%, +25.7%]	**0.005**
*National ADI*							
1st Quartile (1–25)	53.7%	39.5%	53.7%	40.0%	+0.5% [-5.4%, +6.5%]	+1.3% [-0.6%, +3.2%]	0.807
2nd Quartile (26–50)	44.9%	40.3%	56.2%	40.8%	+11.7% [+7.6%, +15.8%]	+0.8% [-1.2%, +2.8%]	**<0.001**
3rd Quartile (51–75)	46.0%	41.0%	53.5%	40.9%	+7.9% [+5.0%, +10.7%]	+0.3% [-2.1%, +2.7%]	**<0.001**
4th Quartile (76–100)	45.3%	41.5%	54.9%	38.4%	+9.9% [+7.2%, +12.6%]	−2.6% [-5.5%, +0.4%]	**<0.001**

*P*-values were generated by comparing the change in adherence rate from 2019 to 2021 at AI-switched sites and the change in adherence rate from 2019 to 2021 at non-AI sites.

Bold values identify statistical significance (*p* < 0.05).

We examined the change in DED testing adherence rate across 8 demographic and social determinants of health categories: gender, age, race, ethnicity, language preference, insurance coverage, geography, and national ADI quartile (Table 2). The largest adherence gaps at AI-switched sites in 2019 had significantly decreased by 2021 after AI implementation. For the race category, the largest initial gap in 2019 was between American Indian or Alaska Native patients and Native Hawaiian or Other Pacific Islander patients. This gap shrank from 37.3% in 2019 to 20.5% in 2021. For the insurance category, the largest initial gap in 2019 was between patients with military insurance and those covered by Medicaid. This gap shrank from 32.9% in 2019 to 21.1% in 2021. For the ADI category, the largest initial gap in 2019 was between the 1st and 2nd ADI quartiles. This gap shrank from 8.8% in 2019 to −2.5% in 2021. From 2019 to 2021, among the AI-switched sites, the largest improvements in adherence rate within the categories of race, insurance coverage and ADI quartile were: +19.0% in Native Hawaiian or Other Pacific Islander patients, +12.2% in Black or African American patients, +13.7% in Medicaid-insured patients, and +11.7% in the 2nd ADI quartile.

## Discussion

In this study, we examined the change in annual DED testing adherence rate before and after implementation of autonomous AI technology at primary care clinics at Johns Hopkins Medicine. From 2019 to 2021, i.e. coming out of the COVID pandemic, which caused widespread adherence issues, we observed a substantial increase in adherence rate among AI-switched sites, while the adherence rate among non-AI sites remained unchanged. This improvement in AI-switched sites over non-AI sites, 7.6 percentage points, remained statistically significant after adjustment by propensity score weighting methods. Among the AI-switched sites, the patient populations that experienced substantial improvement in adherence rate included Black or African American patients, patients with Medicaid insurance coverage, and patients with high ADI scores. Therefore, our data suggests that deployment of autonomous AI improved access to retinal evaluation and health equity in these historically disadvantaged patient groups. Our additional observations are as follows.

First, the overall adherence rate across all sites was 42.2% in 2019, which was lower than the nationwide average of 58.3%^12^, but higher than the 34% seen in other low-income metropolitan populations in the United States^3^. The overall adherence rate in 2021 increased slightly to 44.8%. However, the adherence rate among AI-switched sites substantially increased to 54.5%, much closer to the nationwide average. By extrapolation of our data, large scale deployment of this technology across the entire health system could substantially increase overall adherence rate, which in turn could improve HEDIS metrics, Centers for Medicare and Medicaid Services (CMS) Merit-based Incentive Payment System (MIPS) rating, and payer reimbursement.

Second, among the AI-switched sites, there were significant outsize increases in adherence rates in the Black or African American ( + 12%) and Native Hawaiian or Other Pacific Islander ( + 19%) patient populations from 2019 to 2021. In contrast, over the same time period, the adherence rates for these two patient groups actually decreased by 0.6% and 1%, respectively, among non-AI sites. These data suggest that the deployment of autonomous AI improved access when it comes to DED management, particularly for historically disadvantaged populations. Prior studies have found that the African American population uses eye care services at much lower rates than White patients, even though Black patients with type 2 diabetes are significantly more likely to develop retinopathy than White patients^13–16^. A previous focus group found that for many African American patients, transportation, lack of free time, and inconvenience of eye exam served as main barriers for annual DED screening^16^. In fact, minority populations overall have consistently lower unadjusted eye examination rates than White populations^17^.

Third, our data also suggests that autonomous AI was associated with improved health equity and smaller care gaps between patient groups. Among AI-switched sites and before AI deployment in 2019, a large adherence rate gap existed between Asian Americans and Black/African Americans (61.1% vs. 45.5%). This 15.6% gap shrank to 3.5% by 2021. Similarly, among AI-switched sites and before AI deployment in 2019, a large adherence gap existed between patients with military insurance and patients with Medicaid insurance (63.1% vs. 30.2%). This 32.9% gap shrank to 21.1% by 2021. Lastly, among AI-switched sites and before AI deployment in 2019, an adherence rate gap of 8.4% existed between the most socioeconomically advantaged (ADI 1st quartile) and the most socioeconomically disadvantaged (ADI 4th quartile). By 2021, the adherence rate gap between patients from all 4 quartiles had closed.

Fourth, we observed treatment heterogeneity with improvement in adherence rate even after autonomous AI deployment. Though autonomous AI improved access to retinal evaluation for the most disadvantaged patient groups and reduced care gaps, such improvement was not universal. For example, among the AI-switched sites, multiple patient subgroups barely experienced any improvement in adherence rate from 2019 to 2021: Asian ( + 0.4%), American Indian or Alaska Native ( + 2.3%), non-English speaking patients ( + 2.8%), and military insurance coverage ( + 2.8%). While it is beyond the scope of the current study to evaluate the cause for this treatment heterogeneity, the relative lack of trust in healthcare AI or concerns about data usage by patients could be a limiting factor in adoption^18–20^. Patient trust in autonomous AI technologies will affect adoption of AI into our healthcare system, and this is certainly an important topic that warrants more thorough investigation.

Our study is limited by its retrospective nature and the fact that nearly all patients included in our study live in a metropolitan area, so our observations may not generalize to patient populations living in micropolitan, small town, and rural residences. However, our data demonstrated that deployment of autonomous AI for DED testing in the primary care setting is highly associated with improvement in adherence rate, patient access and health equity. We employed propensity score weighting methods to address the inherent limitations of an observational study and are reassured that our analyses showed a significant impact of autonomous AI on DED testing adherence rates. Future studies in the form of a prospective randomized clinical trial, similar to a recent study that investigated the role of autonomous AI for DED testing in youth with diabetes^21^, could help delineate whether there is a causal relationship between autonomous AI and improvement in population-level metrics. Additionally, qualitative surveys that evaluate patient views toward the use of AI technology could help identify targets of patient education that can help address the treatment heterogeneity observed in our study. The actual follow-up rate with ophthalmology for patients who test positive for more than mild diabetic retinopathy per autonomous AI and the associated impact on healthcare cost should be evaluated and quantified as well.

## Methods

This was a retrospective study approved by the Institutional Review Board of the Johns Hopkins School of Medicine. All research adhered to the tenets of the Declaration of Helsinki. Our study included all patients with diabetes mellitus who were managed at primary care sites of Johns Hopkins Medicine in the calendar years 2019 (pre-AI deployment) and 2021 (post-AI deployment). Subject demographic information that was retrieved from the electronic health records system included gender, age, race, ethnicity, preferred language, insurance status, ZIP code of residence, national area deprivation index (ADI), and inflation-adjusted median household income in the past 12 months.

Additionally, from the ZIP code data, each patient’s residence was identified as metropolitan, micropolitan, small town, or rural based on the 2010 Rural-Urban Commuting Area Codes^22^. The national ADI score is a 100-point scale that measures socioeconomic disadvantage based on data like a geographic region’s housing quality, education, income, and employment^23^. A higher national ADI score correlates with increased overall socioeconomic disadvantage.

Each patient’s clinic site was categorized as either “AI-switched” or “non-AI” site. An “AI-switched” site is defined as a site that did not have autonomous AI DED testing (LumineticsCore® formerly known as IDx-DR, Digital Diagnostics, Coralville, IA) in 2019, but had it by 2021. The same autonomous AI system was deployed across all AI-switched sites. A “non-AI” site is defined as a site that never had autonomous AI deployment and where patients are referred to eyecare for DED testing.

### Statistical analyses

Patient demographic characteristics were described. Categorical and binary variables were reported as numbers and percentages. These variables were compared using chi-squared tests (Table 1).

The primary outcome measure was adherence to annual DED testing in a given calendar year. Adherence was defined as either receiving an autonomous AI exam or a dilated eye exam with an ophthalmologist. To compare the primary outcome between AI-switched sites and non-AI sites, we used propensity score weighting methods to ensure comparability across sites and years. The inverse-probability-weighted regression adjustment model was used to estimate the average treatment effect (ATE) in four groups (non-AI sites in 2019, AI-switched sites in 2019, non-AI sites in 2021, and AI-switched sites in 2021). These four groups were matched based on all the relevant covariates (age, gender, race, ethnicity, preferred language, insurance, ADI quartile, family income, and geographical region), as well as an adjusted Poisson regression with the covariates for the outcome model to estimate the difference in adherence rates. The covariates balance was checked after the inverse probability weighting to ensure appropriate balance on all covariates among the four groups. Robust standard errors estimate clustering on participants was used to account for patients who were seen in both 2019 and 2021. The difference-in-difference was also estimated from this model.

The changes in adherence rates among various patient subgroups were calculated using Poisson regression models with Generalized Estimating Equation framework and are reported as percentages with 95% confidence intervals (Table 2).

Statistical analysis was performed using R Statistical Software Version 4.3.1 (R Foundation for Statistical Computing, Vienna, Austria), and the propensity score weighting methods were carried out in Stata Version 17.0. The level of statistical significance was set at *p* < 0.05.

### Reporting summary

Further information on research design is available in the Nature Research Reporting Summary linked to this article.

### Supplementary information

Reporting Summary

## Data Availability

The original data will be available upon official written request to the corresponding author.

## Code availability

The code used for data analysis will be available upon official written request to the corresponding author.
