# Author Correction: Autonomous artificial intelligence for diabetic eye disease increases access and health equity in underserved populations

**DOI:** 10.1038/s41746-024-01229-y

**Published:** 2024-08-23

**Authors:** Jane J. Huang, Roomasa Channa, Risa M. Wolf, Yiwen Dong, Mavis Liang, Jiangxia Wang, Michael D. Abramoff, T. Y. Alvin Liu

**Affiliations:** 1 Wilmer Eye Institute, Johns Hopkins University School of Medicine, Baltimore, MD USA; 2 University of Wisconsin-Madison School of Medicine and Public Health, Madison, WI USA; 3 Johns Hopkins Pediatric Diabetes Center, Johns Hopkins University School of Medicine, Baltimore, MD USA; 4 Department of Biostatistics, Johns Hopkins University, Baltimore, MD USA; 5 Department of Ophthalmology and Visual Sciences, University of Iowa, Iowa City, IA USA; 6 Department of Electrical and Computer Engineering, University of Iowa, Iowa City, IA USA

**Keywords:** Retinal diseases, Disease prevention, Outcomes research, Diabetes complications, Machine learning

Correction to: *npj Digital Medicine* (2024) **7**:196; 10.1038/s41746-024-01197-3, published online 22 July 2024

In the Acknowledgements section, the funding source “This study was partially funded by the Research to Prevent Blindness Career Advancement Award that supports TYAL” should have read ‘This study was partially funded by the Research to Prevent Blindness / Dr. H. James and Carole Free Career Development Award that supports TYAL.”’. The original article has been corrected.

